# Overcoming cancer drug resistance through small-molecule targeting of HSP90 and HSP70

**DOI:** 10.20517/cdr.2025.149

**Published:** 2025-12-01

**Authors:** Ren-Duan Cai, Ming-Jing Lin, Qing-Mei Ye

**Affiliations:** Hainan General Hospital & Hainan Affiliated Hospital of Hainan Medical University, Haikou 570311, Hainan, China.; ^#^These authors contributed equally to this work.

**Keywords:** Drug resistance, cancer, HSPs, inhibitors, overcome

## Abstract

Heat shock proteins (HSPs) play a critical role in cancer progression and drug resistance by stabilizing oncoproteins, enhancing DNA repair, and modulating apoptosis pathways. In particular, HSP90 and HSP70 have been implicated in maintaining the survival of drug-resistant cancer cells. Consequently, targeting HSPs holds promise in combating drug resistance in cancers. HSP inhibitors induce apoptosis in resistant cancer cells and act as potent chemosensitizers, enhancing the efficacy of chemotherapy, radiotherapy, and targeted therapies. However, despite promising preclinical data, no HSP inhibitors have been approved by the U.S. Food and Drug Administration (FDA) due to toxicity, limited treatment outcomes, or a lack of specificity. In this review, we attempted to provide a brief overview of small-molecule HSP inhibitors, including the medicinal chemistry of geldanamycin derivatives, resorcinol-based compounds, and purine-scaffold inhibitors. We summarized the recent advancements of HSP inhibitors, especially those in clinical trials, their mechanisms of action, and their combinations in overcoming multidrug resistance in cancers. Furthermore, we discussed the current challenges and proposed possible solutions.

## INTRODUCTION

Heat shock proteins (HSPs), also known as stress proteins, are produced by cells in response to elevated temperatures or other stress conditions such as toxins, ultraviolet light, or inflammation. They play critical roles in cellular biology, primarily acting as chaperones that assist in protein folding, prevent aggregation, and aid in protein transport across membranes^[1]^. HSPs belong to several families, each characterized by their molecular weight, with the main groups being HSP100, HSP90, HSP70, HSP60, HSP40, and small HSPs (sHSPs). Specifically, HSP90 and HSP70 are among the most studied in cellular biology and cancer cells^[2]^. Renowned for its role in the maturation of signaling proteins such as kinases and steroid hormone receptors, HSP90 forms a homodimer with an N-terminal adenosine triphosphate (ATP)-binding domain, a middle domain for substrate binding, and a C-terminal dimerization domain, which allows for conformational changes that are crucial for its chaperone function^[2,3]^. HSP70 represents another one of the most studied HSPs. HSP70 has a nucleotide-binding domain (NBD) and a substrate-binding domain (SBD), facilitating the binding to hydrophobic regions of misfolded proteins, which then helps refold or direct them to degradation pathways^[4]^. There is also interaction or cooperation between different members; for instance, HSP40 (J-proteins) works in concert with HSP70, assisting in the binding and delivering substrate proteins to HSP70^[5]^.

**Figure 1:**
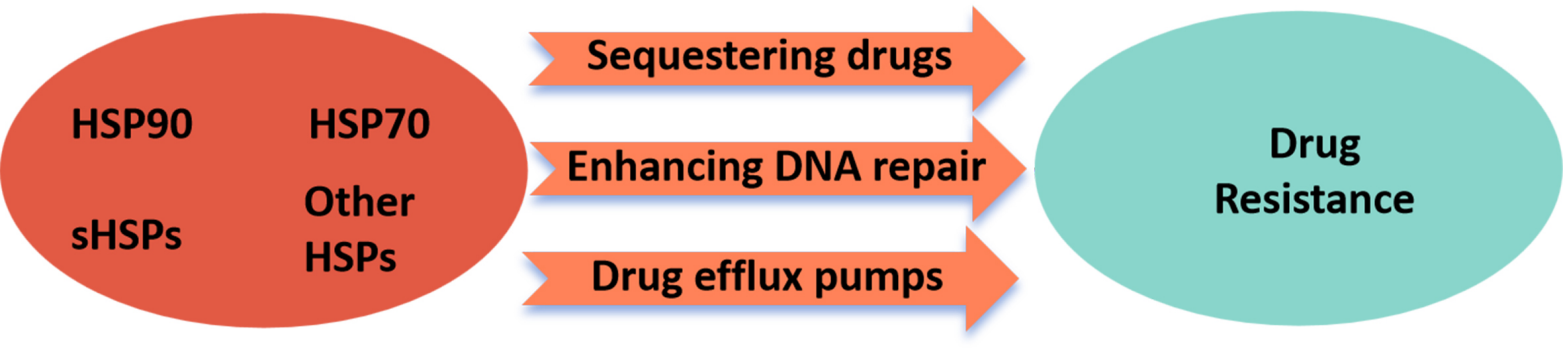
HSPs induce drug resistance in cancers via various mechanisms. HSP90, HSP70, sHSPs, and other HSPs contribute to resistance by sequestering therapeutic agents, stabilizing DNA repair proteins to enhance repair capacity, and regulating drug efflux transporters to reduce intracellular drug accumulation. This figure was generated by the PowerPoint software. HSPs: Heat shock proteins; sHSPs: small heat shock proteins.

Due to their essential functions in protein homeostasis, stress response, and cell signaling, HSPs can be harnessed by cancer cells for their survival and migration. More importantly, they have been validated to induce drug resistance in cancers as summarized in Figure 1^[6,7]^. In general, under the stress of chemotherapy or radiotherapy, cancer cells upregulate certain HSPs to adapt to the harsh or toxic environment, enabling them to survive treatments that would normally be lethal^[8]^. This adaptation can make subsequent treatments less effective, i.e., resistance. Specifically, HSPs are multifaceted in conferring resistance to various chemotherapeutic agents. One key mechanism involves the sequestration of cytotoxic drugs, which effectively reduces their intracellular concentrations and diminishes their ability to reach critical molecular targets^[6,9]^. This drug-buffering effect undermines therapeutic efficacy and contributes to the survival of otherwise vulnerable cancer cells. Additionally, HSPs are closely involved in maintaining cellular proteostasis under stress conditions^[10]^. They actively repair or refold proteins that have been damaged by chemotherapy-induced oxidative or alkylating stress, thereby preserving the functional integrity of essential cellular machinery. Beyond protein repair, certain HSPs are also known to facilitate the repair of DNA lesions caused by chemotherapeutic agents^[11]^. For instance, HSPs can enhance nucleotide excision repair and homologous recombination pathways, which are particularly important in mitigating the DNA-damaging effects of agents such as cisplatin and doxorubicin^[6,12]^. Moreover, specific members of the HSP family, particularly HSP90, are known to stabilize oncogenic proteins that drive tumor progression and therapy resistance. A prominent example is mutated p53, a protein that loses its tumor suppressor function and often gains pro-survival properties^[13]^. HSP90 binding helps stabilize this dysfunctional protein, preventing its degradation and thereby allowing cancer cells to evade apoptosis, even under treatment pressure^[14]^. By maintaining the activity of such client proteins, HSP90 and other HSPs create a cellular environment conducive to drug resistance and tumor survival^[6,9]^. Furthermore, HSPs may also modulate the function of drug efflux pumps such as P-glycoprotein (P-gp), which are often overexpressed in drug-resistant cancers, leading to the expulsion of chemotherapeutic agents from the cell^[15-19]^. The broad chaperone activity of HSPs suggests that resistance to one drug can confer resistance to others, a phenomenon known as multidrug resistance, as the proteins that confer resistance are maintained in a functional state by HSPs. Given their roles, HSPs are being explored as therapeutic targets. Inhibitors of HSP90 or HSP70, for example, have been developed to destabilize oncoproteins, thereby potentially reversing drug resistance in cancers.

In this paper, we provide an overview of the studies on using HSP inhibitors in drug-resistant cancers, especially those in clinical trials conducted in the past decade. We also briefly discuss medicinal chemistry-based structural differences and variations of these compounds. Additionally, we summarize the combination regimens using HSP inhibitors and other conventional chemotherapeutics, radiotherapy, and targeted therapies. The information gathered can direct future cancer research, and drug discovery and development.

## THERAPEUTIC BENEFITS OF HPS INHIBITORS IN DRUG-RESISTANT CANCERS

**Figure 2:**
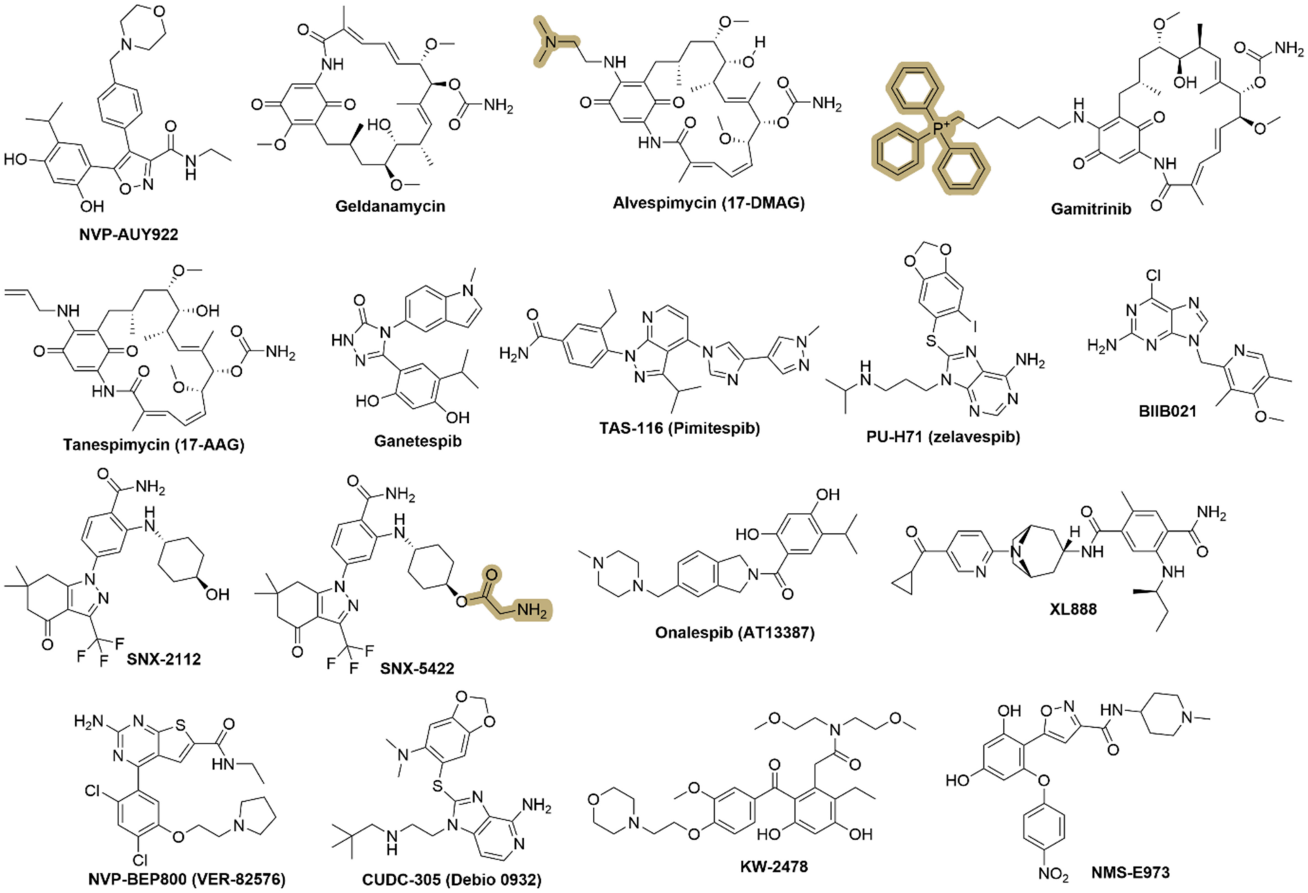
Representative chemical structures of HSP90 inhibitors. Key pharmacophoric features and structural scaffolds are highlighted to illustrate common design strategies and the important functional groups that contribute to HSP90 inhibition. This figure was generated by the ChemDraw Software (V 2020). HSP: Heat shock protein.

**Figure 3:**
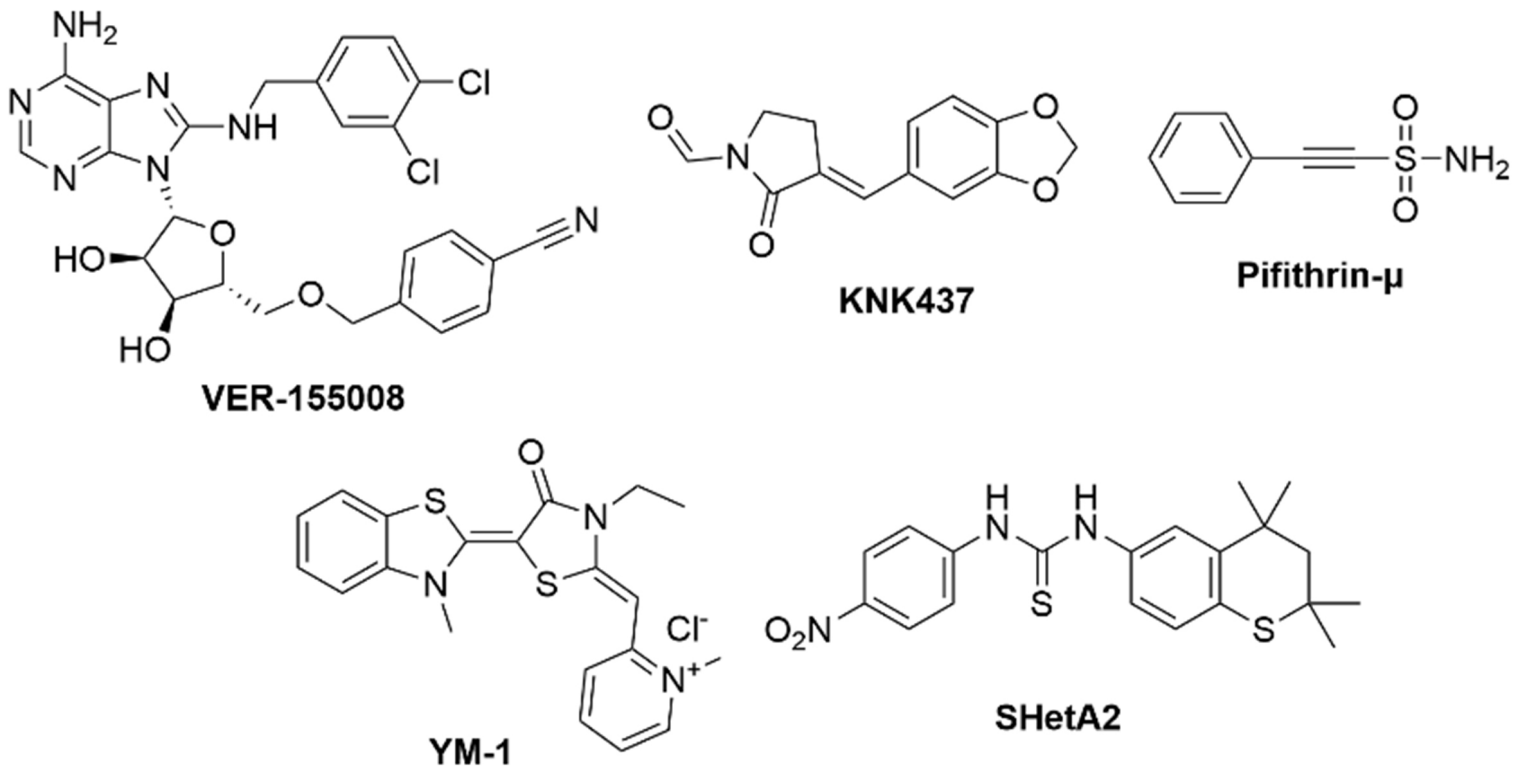
Representative chemical structures of HSP70 inhibitors. These compounds also show potential in drug-resistant cancers. SHetA2 is the only one in a clinical trial. This figure was generated by the ChemDraw Software (V 2020). HSP: Heat shock protein; SHetA2: sulfur heteroarotinoid A2.

To date, no HSP inhibitors have been approved by the U.S. Food and Drug Administration (FDA). In this review, we focus on small-molecule inhibitors of HSPs, with particular emphasis on HSP90 [Figure 2] and HSP70 [Figure 3], which represent the most extensively studied and therapeutically relevant subtypes in cancer drug resistance. We also focus on those currently in clinical trials or preclinical studies, as well as closely related compounds. More importantly, recent studies using HSP inhibitors for treating drug-resistant cancers will also be summarized.

Generally, these inhibitors exert their therapeutic potential through two primary mechanisms: (1) directly inducing cytotoxicity in drug-resistant cancer cells; or (2) enhancing the efficacy of conventional chemotherapies and targeted therapies by sensitizing the tumor cells to these treatments. HSP inhibitors, in particular, appear to function overwhelmingly as chemosensitizers, modulating stress response pathways and disrupting protein homeostasis in cancer cells, thereby rendering them more susceptible to certain treatments. Several prominent inhibitors targeting HSPs and related pathways have been extensively evaluated in clinical trials, demonstrating promising efficacy profiles either as monotherapies or in combination with other agents. These inhibitors will be discussed in detail to highlight their mechanisms of action, clinical relevance, and potential for overcoming drug resistance. Meanwhile, other inhibitors that function through similar mechanisms (though perhaps less studied or still in preclinical development) will be briefly summarized to provide a broader perspective on their potential applications, whether used alone or in synergistic combinations.

### NVP-AUY922

NVP-AUY922, a resorcinylic isoxazole amide derivative, is an HSP90 inhibitor developed by Novartis. NVP-AUY922 binds to the N-terminal ATP-binding pocket of HSP90, preventing the adenosine triphosphatase (ATPase) activity necessary for the chaperone function. This results in the destabilization and subsequent degradation of client proteins by the proteasome^[20,21]^. NVP-AUY922 has been evaluated in several clinical trials to assess its safety, pharmacokinetics, and efficacy in human cancers. While it demonstrated promising results in early-phase studies, particularly regarding client protein degradation and tumor response, further development faced challenges, including issues with toxicity and the need for more specific targeting to reduce side effects^[22]^. NVP-AUY922 has been tested both as a single agent and in combination with other treatments such as radiation or chemotherapy, demonstrating enhanced efficacy in some resistant cancer models.

NVP-AUY922 demonstrates potent anticancer activity against human epidermal growth factor receptor 2 (HER2)-positive and trastuzumab-resistant breast cancer cells. It effectively inhibits proliferation across seven HER2-positive breast cancer cell lines, including trastuzumab-resistant models, with IC_50_ values ranging from 6 to 17 nM^[23]^. Treatment with 0.1-1 μM NVP-AUY922 reduces HER2 and Akt protein levels in sensitive and resistant cell lines. The combination of NVP-AUY922 with trastuzumab significantly enhances growth inhibition in select cell lines (BT474, BT474/Tr, and EFM-192A) but shows no additional benefit with chemotherapy agents, such as docetaxel or cisplatin. However, no *in vivo* validation was included in this study^[23]^. NVP-AUY922 also inhibits the proliferation of malignant pheochromocytoma (PCC), with IC_50_ values of 75 nM (48 h) or 30 nM (72 h)^[24]^. NVP-AUY922 induces apoptosis, decreases HER2 and Akt levels, and modulates phosphoinositide 3-kinase (PI3K)/Akt and mitogen-activated protein kinase kinase (MEK)/extracellular signal-regulated kinase (ERK) signaling pathways. In the *in vivo* model, intraperitoneal (IP) administration of NVP-AUY922 (35 mg/kg) significantly reduces tumor growth in PCC xenografts without affecting body weight^[24]^.

NVP-AUY922 enhances the efficacy of (1) radiotherapy in lung cancer^[25]^, glioblastoma cancer stem cell-like cells^[26]^; (2) Bcl-2 inhibitor ABT-737 in small cell lung cancer^[27]^; (3) PI3K inhibitor omipalisib (GSK458)^[28]^; MEK inhibitor trametinib^[29]^ in non-small cell lung cancer (NSCLC); (4) cytarabine in acute myeloid leukemia (AML) cells^[30]^; and (5) heating therapy in colon cancer cells^[31]^. These findings support further clinical investigation of NVP-AUY922 for resistant cancers.

### Geldanamycin

Geldanamycin is a naturally occurring benzoquinone ansamycin antibiotic that functions as a potent inhibitor of HSP90^[32]^, binding to the ATP-binding pocket of HSP90, blocking its chaperone activity and leading to the degradation of oncogenic client proteins such as HER2, Akt, and Raf-1^[33,34]^. Geldanamycin can act as a sensitizer in combination therapy, e.g., with paclitaxel in ovarian cancer cells^[35]^, docetaxel^[36]^ in ovarian cancer, and photodynamic therapy in prostate cancer^[37]^. As a tool compound, it exhibited strong anticancer effects in preclinical studies; however, its clinical development was hindered by severe hepatotoxicity and poor solubility. To overcome these limitations, several geldanamycin derivatives, such as alvespimycin (17-DMAG) and others, were developed and tested in clinical trials as discussed below.

### Alvespimycin

Alvespimycin (17-DMAG or KOS-1022) is a derivative of geldanamycin, developed to have improved pharmacological properties over its parent compound geldanamycin, including better water solubility, reduced hepatotoxicity^[38]^. It has higher oral bioavailability than geldanamycin due to the introduced hydrophilic N-containing function motif [Figure 2]. Alvespimycin can effectively overcome imatinib resistance in chronic myeloid leukemia (CML) K562-RC and K562-RD cells^[39]^. As a sensitizer, alvespimycin worked with (1) imetelstat in human osteosarcoma cells *in vitro* and *in vivo*^[40]^; and (2) trastuzumab in breast and ovarian cancer patients^[41]^. Clinically, alvespimycin has been evaluated in various phase I and phase II trials for different cancers, such as AML, melanoma, and breast and ovarian tumors^[41,42]^. Early trials showed some promise, with signs of clinical activity in AML where it induced complete remission in a subset of patients. However, its development was eventually halted in 2008 by Kosan Biosciences due to an unfavorable toxicity profile.

### Gamitrinib

Gamitrinib is a geldanamycin derivative possessing a mitochondria-targeting motif, triphenylphosphonium (TPP, highlighted in Figure 2); to achieve selective accumulation in mitochondria^[43,44]^, it can disrupt mitochondrial HSP90 function, leading to mitochondrial dysfunction, increased mitochondrial permeability transition, and apoptosis in cancer cells^[45]^. It has shown promising preclinical activity against advanced prostate cancer, including hormone-refractory and drug-resistant forms, with a first-in-human phase I clinical trial, although details are not disclosed in the available data^[44]^.

Gamitrinib, by inhibiting mitochondrial tumor necrosis factor receptor-associated protein-1 (TRAP-1), a HSP90 chaperone, was able to (1) effectively inhibit metastatic prostate cancer cells^[46]^; (2) suppress the proliferation of glutamine-dependent NSCLC cells^[47]^; (3) enhance the effect temozolomide^[48,49]^ and HDAC1/2 inhibitor panobinostat^[50]^ in glioblastoma multiforme cells; and (4) augment the cytotoxicity of paclitaxel in paclitaxel-resistant breast carcinoma cells^[51]^. Additionally, Gamitrinib could reverse Bcl2-mediated resistance in Hep3B cells^[52]^, and it could also work synergistically with Bcl-2 inhibitors, including ABT263, obatoclax, ABT199, WEHI-539, and A1210477 in a panel of therapy-resistant tumors *in vitro* and *in vivo* in murine model systems of melanoma, triple-negative breast cancer (TNBC), and patient-derived orthotopic xenografts (PDX) of human glioblastoma^[53]^. Furthermore, Gamitrinib also showed potential in MAPK inhibitor-resistant melanoma cell lines^[54]^, and it worked synergistically with doxorubicin in breast and prostate cancer cells *in vitro* and *in vivo*^[55]^.

### Tanespimycin

Tanespimycin, also known as 17-AAG short for 17-allylamino-17-demethoxygeldanamycin, is a synthetic geldanamycin derivative as a HSP90 inhibitor^[56]^. It has been explored in clinical trials for various cancers^[56]^, showing significant activity particularly in combination with trastuzumab in HER2-positive metastatic breast cancer patients who had previously progressed on trastuzumab^[57]^. Despite promising results in phase II trials, including response evaluation criteria in solid tumors (RECIST)-defined responses in solid tumors, the development of tanespimycin was suspended^[57]^.

Similar to its precursor geldanamycin, the leading compound tanespimycin showed strong cytotoxicity against drug-resistant cancer cells, including AML cancer stem cells^[58]^ and castration-resistant prostate cancer cells^[59]^. As a sensitizer, tanespimycin can combine with (1) cisplatin in cisplatin-resistant esophageal squamous cell carcinoma cells^[60]^; (2) proteasome inhibitor bortezomib in patients with advanced solid malignancies^[61]^; and (3) gemcitabine in advanced epithelial ovarian and primary peritoneal carcinoma^[62]^. These studies highlight tanespimycin’s great potential as a lead compound. Further structural optimization is needed to advance its clinical trials and utilization.

### Ganetespib

Ganetespib (STA-9090) is a second-generation, small-molecule HSP90 inhibitor. Unlike geldanamycin-based inhibitors, it is a resorcinol-containing compound that binds to the ATP-binding pocket of HSP90, disrupting its chaperone function and leading to the degradation of oncogenic client proteins. Ganetespib effectively kills resistant cancer cells, including ErbB2-overexpressing breast cancer cells^[63]^, and it sensitizes other chemotherapeutics in various cancer types. Additionally, Ganetespib enhances the sensitivity of mantle cell lymphoma (MCL) cells to the Bruton’s tyrosine kinase (BTK) inhibitor ibrutinib, particularly in resistant cancer cells. Transient treatment with ganetespib (12.8 nM for Jeko-1 and 45.7 nM for Granta-519 cells) for 12 h reduced the IC_50_ of ibrutinib (3.24 μM for Jeko-1 and 5.72 μM for Granta-519 cells) by approximately 2.5-fold. This combination led to enhanced G0/G1 cell cycle arrest, increased apoptosis via caspase-9 activation and BCL-2 downregulation, and greater accumulation of DNA damage. In a Jeko-1 xenograft model, ganetespib (15 mg/kg, weekly) combined with ibrutinib (50 mg/kg, daily) significantly reduced tumor volume by 74.49% compared to the control, with notable decreases in Ki-67 and BCL-2 expression, suggesting that sequential administration of ganetespib and ibrutinib could be a promising strategy for overcoming ibrutinib resistance in MCL^[64]^. In a similar manner, Ganetespib could also enhance the sensitivity of (1) radiotherapy in head and neck cancer cells^[65]^, lung cancer^[66]^; (2) sorafenib in HepG2 cells^[67]^; (3) methotrexate in lung cancer A549 cells^[68]^; (4) lapatinib in refractory HER2-positive breast cancer^[69]^; (5) cyclophosphamide in hematological malignancies such as lymphomas^[70]^; (6) cytarabine in AML^[71]^; and (7) cisplatin in cervical cancer^[72]^.

Ganetespib demonstrated potent preclinical activity against various cancers and it progressed through multiple Phases I, II, and III clinical trials, with notable investigation in NSCLC^[73]^. However, despite promising early results, a Phase III trial (GALAXY-2) in NSCLC failed to meet its primary endpoint, leading to its discontinuation from further clinical development^[74]^.

### TAS-116

TAS-116 (pimitespib), a benzamide derivative, is a selective, orally available HSP90 inhibitor that specifically targets the alpha and beta isoforms of cytosolic HSP90 while sparing the mitochondrial isoform TRAP1^[75]^, thereby exhibiting reduced toxicity, particularly hepatotoxicity, due to its selective binding^[76]^. As a chemosensitizer or synergizer, TAS-116 showed positive results when combined with (1) programmed death-ligand-1 (PD-L1) antibody nivolumab in patients with colorectal cancer and other solid tumors^[77]^; (2) radiotherapy in cervical and lung cancer cells *in vitro* and *in vivo*^[78]^; (3) proteasome inhibitor bortezomib in multiple myeloma (MM)^[79]^; and (4) sunitinib in imatinib-resistant gastrointestinal stromal tumors (GIST)^[80]^.

TAS-116 has undergone multiple clinical trials for advanced cancers, particularly GIST^[81]^ and solid tumors^[82]^. Phase I trials established the maximum tolerated dose and showed partial responses in NSCLC and GIST, with manageable side effects, including gastrointestinal and eye disorders^[82]^. A Phase Ib trial combining TAS-116 with nivolumab demonstrated promising activity in microsatellite-stable colorectal cancer^[77]^. The Phase III CHAPTER-GIST-301 trial confirmed TAS-116’s ability to significantly prolong progression-free survival in refractory GIST cases^[83]^. While approved in Japan for fourth-line treatment of GIST^[84]^, its approval in the U.S. remains pending, and it is currently undergoing a phase I clinical trial for GIST. It is optimistic to say that the potential of TAS-116 can be largely expanded after its further approval in the U.S.

### SNX-2112

SNX-2112, 2-aminobenzamide derivative, is a potent, selective, small-molecule inhibitor of HSP90, which is designed to disrupt its ATPase activity and induce degradation of oncogenic client proteins^[85]^. SNX-2112 binds to the ATP-binding pocket of HSP90^[86]^. SNX-2112 has demonstrated strong preclinical efficacy against various cancers^[87]^, and can effectively kill resistant cancer cells, such as human CML K562/ADR cell line^[88]^. SNX-2112 also served as a chemosensitizer to enhance the sensitivity of 5-FU in Eca109 esophageal cancer cells^[89]^. Its prodrug, SNX-5422 [Figure 2], with an aminoacetyl group, was evaluated in Phase I clinical trials for solid tumors and lymphomas but faced challenges due to dose-limiting toxicities, particularly ocular toxicity^[90]^. While promising, further clinical development has been limited, and alternative HSP90 inhibitors have gained more traction in oncology research.

### SNX-7081

SNX-7081 appears to be a derivative of SNX-2112, which has demonstrated enhanced cytotoxicity in a series of cancer cell lines^[91]^. Structural information on SNX-7081 seems to be unavailable, but it appears effective in sensitizing fludarabine for the treatment of p53-negative chronic lymphocytic leukemia (CLL) by enhancing DNA damage and impairing DNA repair mechanisms^[92]^.

### PU-H71

PU-H71 (zelavespib) is a novel purine-scaffold inhibitor targeting HSP90, targeting the ATP-binding site to disrupt its chaperone function^[93]^. Chemically known as 8-[(6-iodo-1,3-benzodioxol-5-yl)sulfanyl]-9-[3-(propan-2-ylamino)propyl]purin-6-amine, PU-H71 consists of a purine core with substitutions including an iodinated benzodioxolyl group linked via a sulfur atom at the 8-position, and an isopropylaminopropyl group at the 9-position [Figure 2]. It has shown significant therapeutic promise in preclinical models across various cancer types, including TNBC, AML, Ewing sarcoma, and in combination with carbon-ion radiotherapy for osteosarcoma^[94]^. PU-H71 could sensitize radiotherapy in lung cancer and breast cancer cells^[95]^. Its activity has been associated with the downregulation of oncoproteins such as Akt, epithelial growth factor receptor (EGFR), and Raf-1, and it has also demonstrated effectiveness in combination therapies, enhancing the effects of other cancer treatments such as BH3 mimetics and radiation therapy^[95]^. Clinically, PU-H71 has undergone a first-in-human study, where it was administered intravenously to assess its safety, tolerability, and pharmacokinetic profile in patients with refractory solid tumors^[96]^. This study confirmed PU-H71 was well-tolerated without dose-limiting toxicities at the tested doses. Further clinical trials are yet to be initiated.

### BIIB021

BIIB021, a derivative of a 2-aminopurine, is a synthetic, orally available small-molecule HSP90 inhibitor, binding to the ATP-binding pocket of HSP90, inhibiting its ATPase activity and leading to the degradation of client proteins^[97]^. It has shown preclinical and early clinical activity against various cancers, including solid tumors and hematologic malignancies^[98]^. BIIB021 advanced to Phase I and Phase II clinical trials but was eventually discontinued^[99]^. It efficiently suppressed the proliferation of both imatinib-sensitive and -resistant CML cells via the mechanistic target of rapamycin (mTOR)-Unc-51-like kinase 1 (Ulk1) pathway^[98]^ and can sensitize esophageal squamous cell carcinoma to radiotherapy^[100]^.

### Onalespib

Onalespib (AT13387), an isoindole derivative, is a small molecule inhibitor that targets HSP90^[101]^. It shows great potential in working synergistically with (1) paclitaxel in patients with advanced TNBC^[102]^; (2) radiotherapy in A-431 epidermoid carcinoma cells^[103]^, and head and neck squamous cell carcinoma^[104]^; (3) Lutetium 177 (^177^Lu) DOTA-0-Tyr3-Octreotate (^177^Lu-DOTATATE) in neuroendocrine tumors^[105]^; (4) abiraterone acetate in prostate cancer^[106]^; (5) temozolomide against malignant gliomas^[107]^; (6) crizotinib or erlotinib in NSCLC^[108]^; (7) imatinib in patients with GIST^[109]^; (8) cisplatin in pancreatic cancer cells^[110]^, and Bcl-2 inhibitor ABT-263 in breast cancer cells^[111]^. It has been evaluated in clinical trials primarily for its antineoplastic potential, with ongoing Phase I studies focusing on cancers such as breast, lung and fallopian tube carcinoma^[102,112,113]^, alongside one completed Phase II trial, highlighting its relevance in treating various malignant solid tumors with specific biomarker profiles such as estrogen receptor (ER) negative and HER-2 deficient expression.

### XL888

XL888 is an HSP90 inhibitor featuring a tropane core, specifically designed to address the limitations of earlier inhibitors by offering improved potency and selectivity^[114]^. It binds to the ATP-binding site within the N-terminal domain of HSP90, leading to the destabilization and subsequent proteasomal degradation of client proteins. XL888 has shown significant activity in overcoming resistance to BRAF (B-Raf proto-oncogene, serine/threonine kinase) inhibitors in melanoma models by degrading multiple resistance mediators such as platelet derived growth factor receptor beta (PDGFRβ), COT (Cancer Osaka Thyroid/MAP3K8), and insulin-like growth factor 1 receptor (IGF1R), thereby restoring apoptosis and enhancing the efficacy of BRAF-targeted therapies^[115]^. Preclinical studies have demonstrated its effectiveness in both *in vitro* and *in vivo* settings, notably in BRAF inhibitor-resistant melanoma cell lines and xenografts, where it induced apoptosis more effectively than combined MEK/PI3K inhibition^[115]^. In addition, XL888 could enhance the anticancer effects of radiotherapy and doxorubicin in liver cancer cells^[116,117]^. Clinically, it has been assessed in Phase I trials for patients with advanced solid tumors, showing manageable toxicity profiles and evidence of target inhibition^[118]^. A recent phase I study showed that combinations of BRAF inhibitor vemurafenib, MEK inhibitor cobimetinib, and XL888 had significant toxicity in patients with advanced melanoma^[119]^.

### NVP-BEP800

NVP-BEP800 (VER-82576, 2-amino-4-[2,4-dichloro-5-(2-pyrrolidin-1-ylethoxy)phenyl]-N-ethylthieno[2,3-d]pyrimidine-6-carboxamide) is a novel, fully synthetic inhibitor that targets HSP90 by binding to its NH_2_-terminal ATP-binding pocket^[120]^. This compound exhibits potent antitumor activity across various cancer types^[120]^. NVP-BEP800 induces client protein degradation, including key oncogenic proteins such as ErbB2, B-Raf(V600E), Raf-1, and Akt, and promotes HSP70 induction, which is indicative of HSP90 inhibition^[121]^. It has shown significant preclinical efficacy in human tumor cell lines and primary human xenografts at nanomolar concentrations, with notable activity in reducing tumor growth in xenograft models, causing regression in the BT-474 breast cancer model^[121]^. NVP-BEP800 has also demonstrated the ability to sensitize tumor cells to ionizing radiation by impairing the cell cycle, increasing DNA damage, and prolonging DNA repair^[122]^. It is currently in the preclinical phase.

### CUDC-305 (Debio 0932)

CUDC-305 (Debio 0932) is an orally active inhibitor of HSP90, featuring an imidazo[4,5-c]pyridine core and specifically targeting both HSP90α and HSP90β isoforms with IC_50_ values of 100 and 103 nM, respectively^[123]^. This compound belongs to the imidazopyridine class and exhibits unique pharmacologic properties advantageous for cancer therapy^[123]^. CUDC-305 demonstrates the ability to cross the blood-brain barrier (BBB), achieving therapeutic levels in brain tissue. This makes it particularly promising for treating brain malignancies such as glioblastoma^[123]^. Additionally, CUDC-305 has shown dose-dependent antitumor activity in various xenograft models, including U87MG glioblastoma^[123]^, erlotinib-resistant NSCLC^[124]^, TNBC MDA-MB-468 cells^[123]^, and AML MV4-11 cells^[123]^, significantly prolonging animal survival in orthotopic models. By inhibiting HSP90, CUDC-305 triggers the degradation of multiple oncoproteins, inhibits signaling pathways such as PI3K/AKT and RAF/MEK/ERK, induces apoptosis, and enhances the antitumor activity of standard-of-care agents when used in combination studies. As of the information available, CUDC-305 was licensed by Curis, Inc. to Debiopharm Group in 2009 for worldwide development, with plans to initiate a Phase I clinical trial in Fall 2009. However, recent updates on its clinical trial status or further clinical development beyond this point are not explicitly detailed in the available data.

### KW-2478

KW-2478 is a novel, non-ansamycin, non-purine HSP90 inhibitor designed to treat various malignancies^[125]^. It has shown significant antitumor activity, particularly in MM and B-cell malignancies^[126]^. KW-2478 acts by binding to the ATP-binding site of HSP90^[125]^. In preclinical models, it has demonstrated synergistic effects when combined with bortezomib, enhancing the inhibition of cell proliferation and inducing apoptosis in MM cells, including those resistant to other treatments^[127]^. It also synergizes with cisplatin to inhibit colorectal cancer cells *in vitro* and *in vivo*^[128]^. Clinically, KW-2478 has been evaluated in Phase I/II studies, demonstrating safety, tolerability, and efficacy in patients with relapsed or refractory MM when combined with bortezomib^[129]^. The drug was well-tolerated, with no dose-limiting toxicities observed up to the highest tested doses, and it notably did not manifest significant retinal or ocular toxicity, a concern with some HSP90 inhibitors. The recommended Phase II dose (RP2D) was established, with an objective response rate (ORR) of 39.2% in the efficacy-evaluable population^[129]^. However, detailed updates on further clinical progress or current status beyond these trials are not explicitly stated in the available data.

### NMS-E973

NMS-E973 is a potent, selective inhibitor of HSP90 that belongs to the isoxazole-derived class^[130]^. It binds to the ATP-binding site of HSP90α with subnanomolar affinity, showing high selectivity against kinases and other ATPases^[131]^. This compound has demonstrated significant antiproliferative activity across various tumor cell lines and possesses a favorable pharmacokinetic profile, including the ability to cross the BBB^[132]^. Additionally, NMS-E973 has been shown to induce tumor shrinkage in different human tumor xenografts and is particularly effective in models of resistance to kinase inhibitors, showcasing its potential against drug-resistant cancers^[133]^. Its efficacy extends to intracranial tumor models, indicating promise in treating brain metastases^[133]^.

Following the discussion of HSP90 inhibitors, we would next like to turn to HSP70, another key chaperone that supports protein folding, stability, and stress-response pathways. Targeting HSP70 offers a complementary strategy, particularly in cancers resistant to HSP90 inhibition. In the next section, we briefly summarize recent advances in HSP70 inhibitors, including both novel and repurposed compounds, as illustrated in Figure 3.

### VER-155008

VER-155008 is a novel adenosine-derived inhibitor specifically targeting HSP70, with an IC50 of approximately 0.5 μM^[134]^. It inhibits HSP70 by binding to its ATPase domain^[135]^. VER-155008 also binds to other members of the Hsp70 family, such as Hsc70 and Grp78, but with less affinity^[136]^. This compound has shown the ability to inhibit cell proliferation and synergize with bortezomib, photothermal therapy, and microtubule-targeting agents in multiple human tumor cell lines, including myeloma and colon, breast, and lung cancers, and to induce both caspase-dependent and -independent apoptosis^[137,138]^.

### KNK437

KNK437 is a small-molecule benzylidene lactam derivative that acts as an inhibitor of HSP expression of both HSP70 and HSP90^[139]^. KNK437 sensitizes cancer cells to stress-induced apoptosis, including heat shock, radiation, and chemotherapy arsenic trioxide and gemcitabine^[140,141]^. KNK437 has been widely used as a research tool to study HSP-related mechanisms in cancer and stress biology, but it has not advanced to clinical trials.

### Pifithrin-μ

Pifithrin-μ (PFT-μ), a p53 inhibitor that can bind to mitochondria, has been repurposed to target the inducible form of HSP70, interfering with its carboxyterminal SBD to disrupt its association with client proteins^[142]^. This inhibition can enhance the antitumor effects of various treatments such as hyperthermia, chemotherapy, and radiation by reducing the protective stress response in cancer cells, thereby increasing their sensitivity to these therapies^[143]^. PFT-μ has demonstrated efficacy in preclinical models against multiple cancer types, including prostate, lung, pancreatic, liver, and leukemia cells, showing synergy with other anticancer agents such as cisplatin, cytarabine^[144]^, and sorafenib^[145]^. It is still in the laboratory research or preclinical phase, with ongoing studies to explore its therapeutic potential further.

### YM-1

YM-1 is an allosteric inhibitor of HSP70 that binds within the nucleotide binding domain to stabilize HSP70 in its ADP-bound state, thereby inhibiting ATP turnover and protein refolding^[146]^. Its structure, featuring a benzothiazole moiety, 2-((Z)-((E)-3-ethyl-5-(3-methylbenzo[d]thiazol-2(3H)-ylidene)-4-oxothiazolidin-2-ylidene)methyl)-1-methylpyridin-1-ium chloride, which contributes to its unique interaction with HSP70 [Figure 3]^[147]^. YM-1 blocks the interaction between HSC70 and Bag1, enhancing the binding of HSP70 to misfolded proteins^[147]^. YM-1 exhibits anticancer activity by destabilizing oncoproteins such as Akt and Raf-1, demonstrating efficacy against various cancer cell lines with similar EC_50_ values, e.g., tamoxifen-resistant MCF7 breast cancer cells^[146]^. Its ability to synergize with other treatments by increasing cancer cell susceptibility to apoptosis makes it a promising candidate for combination therapies.

Of note, drugs that bind the ATP-binding domains of HSPs often exhibit inherent toxicity, as these domains are highly conserved across many cellular proteins, leading to unintended interactions with multiple off-target ATP-binding proteins. This lack of selectivity has been a significant limitation in the clinical development of ATP-competitive HSP inhibitors.

In contrast, the small-molecule HSP70 inhibitor sulfur heteroarotinoid A2 (SHetA2) represents a different strategy by interacting with HSP70 family proteins other than those encoded by the *HSPA1* gene, such as mortalin encoded by the *HSPA9* gene, and targeting the SBD of mortalin^[148]^ rather than the ATP-binding pocket^[149]^. This unique binding mode has been associated with a more favorable toxicity profile. SHetA2 has advanced into clinical evaluation, where it is being investigated in a Phase I clinical trial (NCT04928508) for patients with advanced solid tumors. Early results indicate that SHetA2 is well tolerated, and exhibits preliminary signals of antitumor activity. More combination regimens are warranted to investigate the reversal of drug resistance.

## DISCUSSION

### Summary

Drug resistance remains a leading cause of cancer treatment failure and mortality, posing a persistent and formidable challenge that necessitates extensive basic research and translational and clinical investigations^[18,19,150-152]^. Among the diverse strategies explored to overcome this barrier, targeting molecular chaperones, particularly HSPs, has emerged as a promising approach^[153]^. In this review, we highlight the potential of HSP inhibitors as both direct cytotoxic agents and sensitizers in the management of drug-resistant cancers. HSP90 and HSP70, two of the most extensively studied members of the HSP family, play central roles in maintaining proteostasis and stabilizing numerous oncogenic client proteins, thereby facilitating the survival of cancer cells under therapeutic stress^[3]^. Inhibiting these chaperones not only disrupts critical signaling pathways essential for the persistence of resistant phenotypes but also enhances the efficacy of existing therapeutic modalities^[154]^. Specifically, HSP inhibition can lead to apoptosis in drug-resistant cancer cells and, at the same time, restore sensitivity to chemotherapy, radiotherapy, and targeted therapies. By integrating direct tumoricidal effects with the ability to modulate resistance mechanisms, HSP inhibitors represent a versatile class of agents with significant translational potential in overcoming multidrug resistance and improving clinical outcomes.

### Key challenges

However, despite the significant progress made in targeting HSPs to combat drug resistance in cancer, several critical challenges and opportunities remain. The first one lies in the lack of specificity of many currently available HSP inhibitors. Most first- and second-generation compounds exhibit broad activity against multiple HSP isoforms or interact with other molecular chaperones, often resulting in undesirable systemic toxicity and limiting their clinical utility^[155]^. Second, cancer cells frequently develop compensatory resistance mechanisms, such as the upregulation of other chaperones or stress response pathways, which may undermine the effectiveness of HSP inhibition. Third, there are clinical translation barriers that continue to hinder the success of HSP inhibitors, including toxicity, limited bioavailability, and challenges in drug delivery, which collectively restrict the achievement of therapeutically effective concentrations in patients^[155]^.

### Future perspectives

To move HSP inhibitors into clinical settings, several directions should be pursued. The first one is to develop more selective inhibitors that can specifically target HSP90, HSP70, or even specific isoforms, thereby reducing off-target effects and enhancing therapeutic efficacy. Second, to address potential resistance issues, future research should also focus on rational drug design, structure-guided optimization, and biomarker-guided strategies that enable better patient stratification and prediction of therapeutic response. The third direction is to explore more combination therapy strategies. Given the strong chemosensitizing effects of HSP inhibitors, integrating them with conventional chemotherapies, targeted therapies, or immunotherapies offers an opportunity to enhance treatment responses, particularly in resistant cancers. Rationally designed regimens, e.g., combining HSP90 inhibitors with BTK inhibitors, PI3K/AKT/mTOR inhibitors, or immune checkpoint inhibitors as we discussed above, warrant deeper evaluation in both preclinical and clinical settings. In addition, recent advances such as HSP-targeting proteolysis-targeting chimeras (PROTACs), which enable the selective degradation of chaperone proteins^[156,157]^, and nanomedicine-based delivery systems, which enhance bioavailability and tumor selectivity^[158]^, represent promising strategies to address the limitations of earlier generations of inhibitors. These innovative strategies may help reduce systemic toxicity, overcome resistance mechanisms, and expand the therapeutic window of HSP-targeted therapies.

Finally, more clinical trials are necessary to translate these promising approaches into effective treatments. Although several HSP inhibitors have advanced into Phase I and II trials, the lack of FDA-approved agents highlights the need for more rigorous validation, biomarker-driven patient selection, and optimization of therapeutic regimens. Future studies should not only refine dosing schedules, identify and overcome resistance mechanisms, and evaluate long-term safety and efficacy, but also incorporate novel modalities such as PROTAC-based degraders and nanotechnology-enabled drug delivery platforms. By addressing these challenges and integrating recent advances, HSP-targeting strategies hold strong potential to become clinically impactful therapies for cancers, and especially drug-resistant cancers.

## CONCLUSION

Growing evidence supports the pivotal role of HSPs in mediating anticancer drug resistance, making them feasible and compelling targets for therapeutic intervention. Small-molecule inhibitors targeting HSP70, HSP90, and other chaperones have opened new avenues for overcoming resistance in various cancer types. While current clinical success remains limited, ongoing advancements in medicinal chemistry, drug delivery systems, and combination strategies offer new hope. As our mechanistic understanding of HSPs deepens, their modulation is likely to emerge as a cornerstone in the fight against drug-resistant cancers. Continued multidisciplinary efforts are warranted to translate these findings into clinically effective therapies that can benefit patients facing drug-resistant disease.

## DECLARATIONS

### Copyright

© The Author(s) 2025.
